# The daily practice of direct oral anticoagulant use in patients with atrial fibrillation; an observational cohort study

**DOI:** 10.1371/journal.pone.0217302

**Published:** 2019-06-06

**Authors:** Anouk J. W. Gulpen, Hugo ten Cate, Yvonne M. C. Henskens, René van Oerle, Rick Wetzels, Simon Schalla, Harry J. Crijns, Arina J. ten Cate-Hoek

**Affiliations:** 1 Thrombosis Expert Center Maastricht and Laboratory for Clinical Thrombosis and Hemostasis, Maastricht, the Netherlands; 2 Cardiovascular Research Institute Maastricht (CARIM), Maastricht, the Netherlands; 3 Department of Internal Medicine, Maastricht University Medical Center, Maastricht, the Netherlands; 4 Central Diagnostic Laboratory, Maastricht University Medical Center, Maastricht, the Netherlands; 5 Department of Cardiology and Radiology, Maastricht University Medical Center, Maastricht, the Netherlands; Inselspital Universitatsspital Bern, SWITZERLAND

## Abstract

**Background:**

Direct oral anticoagulants (DOACs) are administered in fixed doses without monitoring. There is still little published data on the impact of the absence of monitoring on adherence to medication and stability of DOAC plasma levels over time.

**Objectives:**

To explore adherence and stability of DOAC plasma levels over time in patients with atrial fibrillation (NVAF) recently started on DOAC therapy.

**Patients and methods:**

A prospective observational cohort study with structured follow up including assessment of adherence to medication, plasma levels at baseline, 3,6 and 12 months and adverse events.

**Results:**

We included 164 patients; 89% were previous users of a vitamin K antagonist (VKA). One-year adherence was reasonably good: Morisky adherence measurement scores of 6–8 in 92%. The majority of DOAC plasma levels were within reported on-therapy ranges; dabigatran (median 104.4 ng/ml, IQR 110.2), rivaroxaban (median 185.2 ng/ml, IQR 216.1) and on average levels were not different for full and adjusted doses. There was significant variation between patients, but no significant differences over time within individuals. A substantial proportion of patients starting in the upper-or lower 20^th^ percentiles remained there during the entire follow up. Seventeen bleedings (16 minor, 1 major) were reported, no ischemic events and bleeding or thrombotic events were not associated with DOAC plasma levels.

**Conclusions:**

Adherence was reasonably good in the majority of patients. Our data confirm the stability of DOAC plasma levels over time. Knowledge of such data may, in the individual patient, contribute to optimal drug and dose selection.

## Introduction

Direct oral anticoagulants (DOACs) are increasingly used for stroke prevention in patients with non-valvular atrial fibrillation (NVAF). DOACs are at least as effective as vitamin k antagonists (VKA) and associated with less fatal and intracranial bleeding[1–4]. All DOACs are prescribed in fixed daily doses without the need for frequent laboratory-guided adjustment, with regular doses for patients without risk factors for bleeding and adjusted, lower doses for patients with reduced creatinine clearance, increased age or other risk factors for bleeding[5]. Due to their predictable pharmacokinetic and pharmacodynamic profiles, DOAC plasma levels will remain fairly stable within patients over the time, but high interindividual variability in plasma levels has been shown with all DOACs in defined study populations. However, in daily life patients experience changes in health status, variations in co-medication and problems with adherence, with reported non-adherence rates between 17% and 34% in patients with NVAF, which were similar to the warfarin discontinuation rates[6]. This may have substantial impact on the stability of DOAC plasma levels, potentially changing the efficacy and safety of anticoagulant management. Knowledge of variation in DOAC plasma levels in daily life may have impact on the setup of longterm follow up, which also receives increasing attention in international guidance documents[7]. Recommendations include at least annual monitoring of adherence, side effects, complications and blood tests for hemoglobin, renal and hepatic function; however, DOAC plasma level testing is not recommended. While an annual checkup may be sufficient in general, it may be insufficient in conditions such as illness, diminished adherence or fragile elderly subjects. The aim of this prospective cohort study was to assess the stability of DOAC plasma levels over time in relation to patient characteristics, outcomes and adherence to medication in patients with NVAF recently started on a DOAC. Secondary, we checked the prescribed doses in patients compared to the Dutch guidelines and the influence on DOAC plasma levels.

## Materials and methods

### Patients

All patients from the outpatient clinic of the department of cardiology of the Maastricht University Medical Center (MUMC) who started on, or switched to, a DOAC between September 2009 and July 2016 for prevention of systemic embolism in NVAF were eligible for inclusion in this study cohort. There were no exclusion criteria. Patients were asked to participate in the structured follow up study. Patients started with dabigatran (150 or 110 mg) or rivaroxaban (20 or 15 mg); only few patients started on apixaban (5 or 2.5 mg) or edoxaban (60 or 30 mg), Patients using DOAC for other indications than NVAF were not included. Those patients not consenting to participate in the structured follow up but consenting to a single visit provided baseline data only.

### Measurements

Patients were assessed for any adverse event within 1 month of DOAC therapy and at 3,6 and 12 months thereafter. Assessment included information on any bleeding or thromboembolic event, assessed and defined according to the Dutch Thrombosis service guidelines based on the ISTH-criteria[8]. In addition, medication adherence was assessed using the validated 8-item Morisky Medication Adherence Scale (MMAS-8) translated to the Dutch language[9]. The scale consists of eight questions, first seven items having a dichotomous answer (yes/no) that indicates adherent or non-adherent behavior. For item 8, a patient can choose an answer on a 5-point Likert scale, expressing how often happens that a patient does not take his medications. Scores were calculated as stated in the original reports[9–11] and performed at all study visits. Scores obtained from this scale range from 0 to 8 and higher scores indicate better medication adherence. For each patient, an average MMAS-8 score was calculated from the individual MMAS-8 scores at each time point. Scores of 8, 6 to less than 8, and scores of less than 6, were classified as high, medium and low adherence, respectively. Blood was collected for measurement of plasma DOAC levels at all follow-up visits between 9 and 11.30 am. Blood was drawn with minimal stasis in a 3.2% citrated vacuum tube (Becton Dickinson) via a venepuncture of the antecubital vein. Blood was centrifuged in a two-step procedure. First, it was centrifuged for 5 minutes at 2500g at room temperature. For the second step, plasma was centrifuged for 10 minutes at 10000 g at 18°C. The Diluted TT Hemoclot (Hyphen Biomed) test was used for the measurement of dabigatran plasma levels and anti Xa activity DiXal (Hyphen Biomed) for the measurement of rivaroxaban levels. Dabigatran and Rivaroxaban calibrators and assay controls were retrieved from Hyphen Biomed. All measurements were performed according to the manufacturer’s instructions without modifications. The cut-off for a clinically relevant detection limit was set at ≥30 ng/mL for both assays. In addition, renal function was assessed at the start of therapy and after 6 and 12 months.

### Guidelines

Dosing guidelines are region specific; in our study we used the evidence-based recommendations for DOAC in the Netherlands who are based on the ESC guideline for NVAF[7] and phase III studies[1–4]. The following applied: for dabigatran, patients aged 80 years and older, or patients with concomitant verapamil treatment should be treated with an adjusted dose of dabigatran, 110 mg capsule bd. The 110-mg dose should also be considered in patients aged 75–80 years of age, with an estimated MDRD 30–50 ml/min, in case of gastro- esophageal reflux disease (GERD), and/or in case of another anticipated higher bleeding risk. For rivaroxaban, dose should be adjusted (15 mg od) for patients with mild renal failure (30–49 ml/min), or severe renal failure (15–29 ml/min).

### Ethics

The study complies with the principles and requirements of the Declaration of Helsinki and was approved by the Institutional Review Board of the MUMC (METC azM/UM: 11.4.069). All patients provided written informed consent.

### Analysis

For matters of comparison, baseline data are presented for patients who consented to the structured follow up as well as for patients who provided baseline data only but declined to be followed. Continuous data are expressed as mean and standard deviation (SD) for normally distributed data, or as median and interquartile range for non-normally distributed data. T-test for independent samples was performed for comparison of means in case of normally distributed data, otherwise the Mann-Whitney U test was used. Categorical variables are expressed as counts and percentages. Categorical variables were compared using the χ^2^-test or Fisher’s exact test when expected frequencies were <5. Adherence was calculated at 5 points in time and is expressed as percentages of the total group. Associations of study variables with the MMAS-8 were checked with the χ^2^-test. For DOAC levels a geometric mean (GM) with 95% confidence interval (CI) was calculated. Repeated measurements ANOVA or Kruskall Wallis tests, as appropriate, were used to analyse differences in plasma levels over time, outcomes were corrected for multiple testing (Bonferroni). Trajectories were plotted to determine the stability of DOAC-levels within patients with a given dosing regimen. The 20^th^ and 80^th^ percentile of the geometric mean and the number of patients whose levels remained within these ranges, were calculated at each point in time. Univariate associations between bleeding events and the patient characteristics were assessed using the χ^2^-test. Dichotomized values for HASBLED[12] (cut off ≥3) and age (cut off ≥75) were used to create binominal variables. Variables with a p-value of <0.2 in the univariate analysis were entered in a multivariable model. Incidence rates were calculated as number of events per 100 patient-years. For all analyses a p-value of ≤ 0.05 was considered statistically significant. All statistical analyses were carried out using the IBM SPSS Statistics version 23. For graphical purposes, GraphPad Prism (Version 7.0; GraphPad Software Inc.) was used.

## Results

### Patient characteristics

318 NVAF patients were asked for inclusion in the observational follow up study. Of those, 164 consented to be followed in a structured manner, while 154 provided baseline data but refused further follow up. The baseline characteristics of all patients are shown in Table 1. Patients in the follow-up group were younger [69.7 (46–91) vs. 72.0 (41–89) years of age; p = 0.02], more often male (67.1% vs.56.5%; p = 0.03) and had more often a history of bleeding (15.2% vs. 7.8%; p = 0.03). Rivaroxaban was prescribed in 195 (61.3%) patients, dabigatran in 100 (31.4%), apixaban in 21(6.6%) and edoxaban in 2(0.6%). Furthermore, 294 (92.5%) patients previously used VKA, 21(6.6%) received concomitant anti-platelet therapy and 49 (15.4%) had a history of malignancy. For further analyses, only the data for the 158 patients who consented to be followed and who received dabigatran or rivaroxaban were used, because the limited availability of apixaban and edoxaban at the time of the study, the proportion of patients on apixaban and edoxaban was too small to allow meaningful analysis.

**Table 1:** Baseline characteristics for all patients with NVAF.

Characteristic	Follow-up group (N = 164)	Non-follow-up group (N = 154)	P-value
**Age (year) (range)**	69.7 (46–91)	72.0 (41–89)	0.019*
**Male sex n (%)**	110 (67.1)	87 (56.5)	0.033*
**CHA_2_DS_2_-VASC score median (IQR)**	2.14 (1.29)	2.38 (1.30)	0.185
**Previous VKA treatment n (%)**	146 (89)	148 (96)	0.116
**Antiplatelet therapy n (%)**	11 (6.7)	10 (6.5)	0.903
**Non-steroidal anti-inflammatory drugs n (%)**	6 (3.7)	4 (2.6)	0.568
**Malignancy n (%)**	23 (14.0)	26 (16.9)	0.545
**Previous stroke n (%)**	26 (15.9)	21 (13.6)	0.516
**Prior myocardial infarction n (%)**	17 (10.4)	24 (15.6)	0.203
**Prior bleeding n (%)**	25 (15.2)	12 (7.8)	0.031*
**DOAC n (%)**			
**Dabigatran normal dose (150 mg bid)**	28 (17.1)	31 (20.1)	
**Dabigatran reduced dose (110 mg bid)**	22 (13.4)	19 (12.3)	
**Rivaroxaban normal dose (20 mg od)**	98 (59.8)	69 (44.8)	
**Rivaroxaban reduced dose (15 mg od)**	10 (6.1)	18 (11.7)	
**Apixaban normal dose (5 mg bid)**	5 (3.0)	9 (5.8)	
**Apixaban reduced dose (2.5 mg bid)**		7(4.5)	
**Edoxaban normal dose (60 mg od)**	1 (0.6)		
**Edoxaban reduced dose (30 mg od)**		1 (0.6)	
**Renal function at start**			0.639
**Poor renal function (<30 mL/min) n (%)**	2 (1.2)	1 (0.6)	
**Mild renal failure 31–49 mL/min) n (%)**	10 (6.1)	18 (11.7)	
**Normal kidney function (>50mL/min) n (%)**	140 (85.4)	96 (62.3)	
**Missing**	14 (8.5)	44 (28.6)	

### Inter- and intra-individual DOAC plasma levels

**Fig 1:**
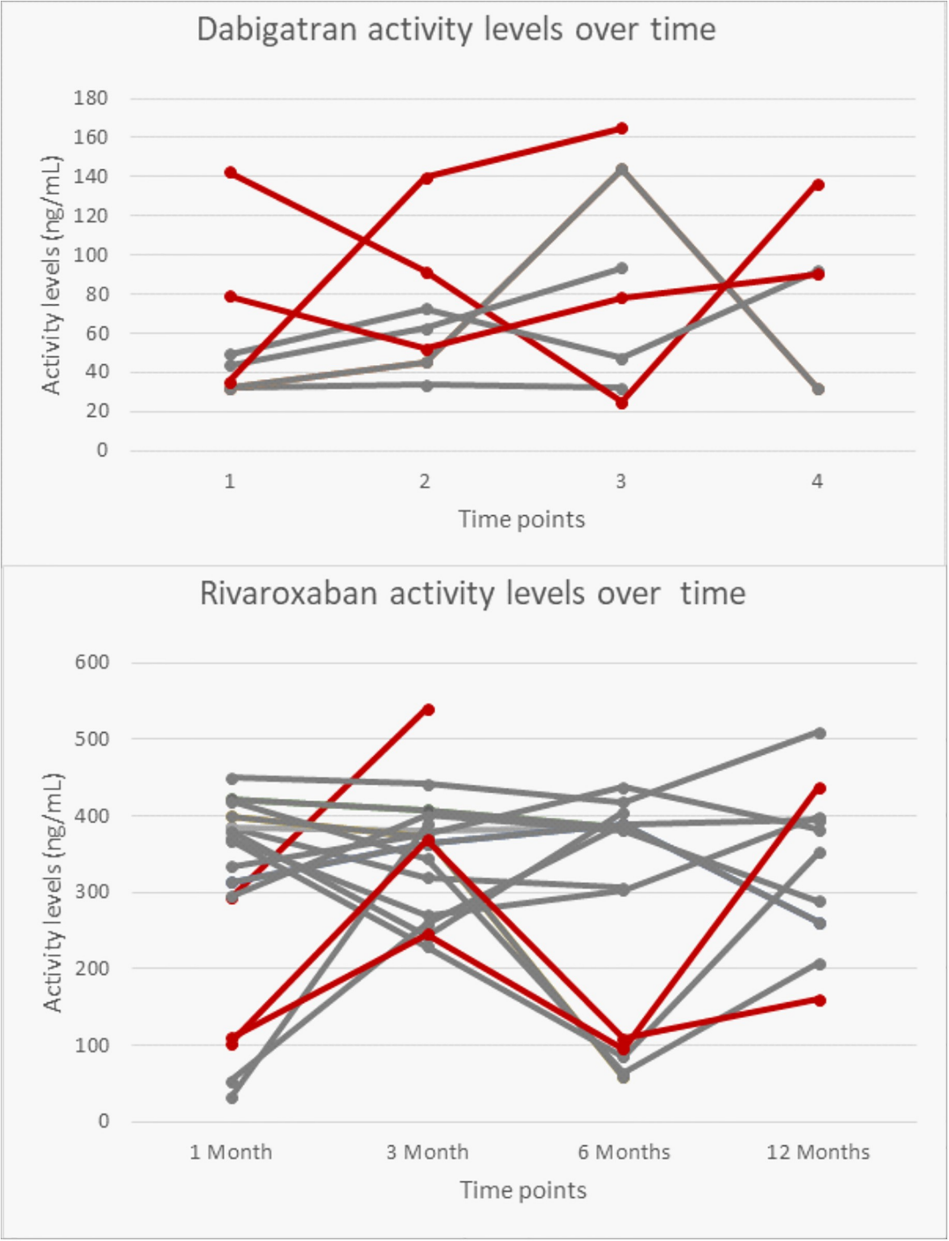
Trajectories for activity levels for patients on dabigatran (upper panel) and rivaroxaban (lower panel) who had one or more data points outside the 20th or 80th percentile. The grey lines represent patients with more than one outlier; the red lines represent patients with 1 outlier.

Table 2 shows the inter-individual plasma levels of the different DOACs. Levels of DOAC were assessed at 1, 3, 6 and 12-months following the onset of therapy. Overall, there were no significant differences over time for the 4 measurements for any of the DOACs tested. For dabigatran 150 mg, plasma levels varied over time from 25 ng/mL to 356.3 ng/mL, (p = 0.577) and for dabigatran 110 mg from 32 ng/mL to 390.8 ng/mL (p = 0.537) Fig 1a and 1b show the trajectories plotted to determine the stability of DOAC-levels within patients with a given dosing regimen for patients on dabigatran and rivaroxaban. Raw data are shown (S1 Table).

**Table 2:** Plasma levels for different doses of DOACs at different time points.

DOAC	1 month (GM, 95% CI)	3 months (GM, 95%, CI)	6 months (GM, 95% CI)	12 months (GM, 95% CI)
**Dabigatran 150 mg bid**	84.9 (54.0–133.6)	84.7 (60.1–119.3)	100.5 (64.6–156.3)	101.2 (73.3–139.9)
**Dabigatran 110 mg bid**	125.4 (77.6–202.7)	114.3 (69.8–187.1)	87.7 (51.2–150.3)	118 (67.9–205.1)
**Rivaroxaban 20 mg od, morning intake**	191.7 (142.1–258.5)	185.7 (145.6–236.9)	219.9 (174.5–277.2)	221 (170–287.5)
**Rivaroxaban 20 mg od, evening intake**	110.6 (81.4–150.1)	117.6 (93.2–148.4)	117.9 (91.7–151.6)	151.1 (115.8–197.1)
Rivaroxaban 15 mg od (GM) combined morning/evening intake*	200.8	91.1	207.1	133.7

For patients taking rivaroxaban 20 mg in the morning (drug dose taken before 11.00 am), mean levels over time were 251.1 ng/mL (SD 123.2). For those taking this drug in the evening (drug dose taken before 21.00 pm), the mean level was 117.1 ng/ml (SD 38.9). Plasma levels for morning and evening intake were similar over time for the entire group (p 0.715). For rivaroxaban 15mg, numbers were too small to perform a separate analysis for timing of intake.

### Plasma levels of DOAC in relation to dosing

Mean levels for the tested DOACs were within recommended on-therapy ranges both for dabigatran and rivaroxaban (Table 2)[13–15]. Mean levels of adjusted doses were comparable to those of the normal dose at all points in time.

Since the mean plasma levels for the normal and reduced dose were not significantly different, we pooled the available data per DOAC to assess the outliers over time and calculated as upper and lower 20^th^ percentiles according to Chan et al[16] (Fig 1). We indicated on-therapy ranges from previously published peak and trough levels derived from patients with NVAF studied in clinical trials[13–15]; this resulted in a range of 30 to 450 ng/mL for dabigatran 150 mg and 40 to 300 ng/mL for dabigatran 110 mg. For rivaroxaban 20 mg, a range of 9 to 361 ng/mL was applied.

Data on plasma levels after one-month were available for 25 patients on dabigatran. For 8 out of 25 (32%) dabigatran patients who had a DOAC level below the 20^th^ percentile at 1 month, the DOAC levels remained below this threshold, for 5 out of 8 at 3 months, and for 2 out of 8 at 6 and 12 months. For 6 out of 25 (24%) dabigatran patients who had DOAC levels above the 80^th^ percentile at 1 month, the DOAC levels remained above this threshold in 4 out of 6 at 3 months, and for 2 out of 6 at 6 months, in none at 12 months.

Data on plasma levels after one-month were available for 73 patients treated with rivaroxaban. For 18 out of 73 (24.7%) rivaroxaban patients who had DOAC levels below the 20^th^ percentile at 1 month, the DOAC levels remained below this threshold in 12 out of 18 at 3 months, in 8 out of 18 at 6 months and in 3 out of 18 at 12 months. For 18 out of 73 (24.7%) patients who had a DOAC level above the 80^th^ percentile at 1 month, this plasma level remained above this threshold in 8 out of 18 at 3 months, in 9 out of 18 at 6 months and in 4 out of 18 at 12 months.

In addition, also patients starting out within the normal on therapy range showed plasma levels outside this range at some point in time during follow up. Therefore, we also calculated the total number of values in the upper and lower 20^th^ percentiles at any point in time during follow up.

For dabigatran, there were an additional 3 patients below the 20^th^ percentile after 1 month, another 2 after 3 months, 3 after 6 months and 3 patients after 12 months and additionally 2, 1 and 5 patients above the 80^th^ percentile after 3,6 and 12 months respectively.

For rivaroxaban, also some patients who started out within the 20-80-percentile range acquired plasma levels outside these ranges; there were an additional 10 patients below the 20^th^ percentile after 3 months, 12 patients after 6 months and two after 12 months. Also, above the 80^th^ percentile additional patients were identified: 10 after 3 months, 6 after 6 months, and 6 patients after 12 months.

**Fig 2:**
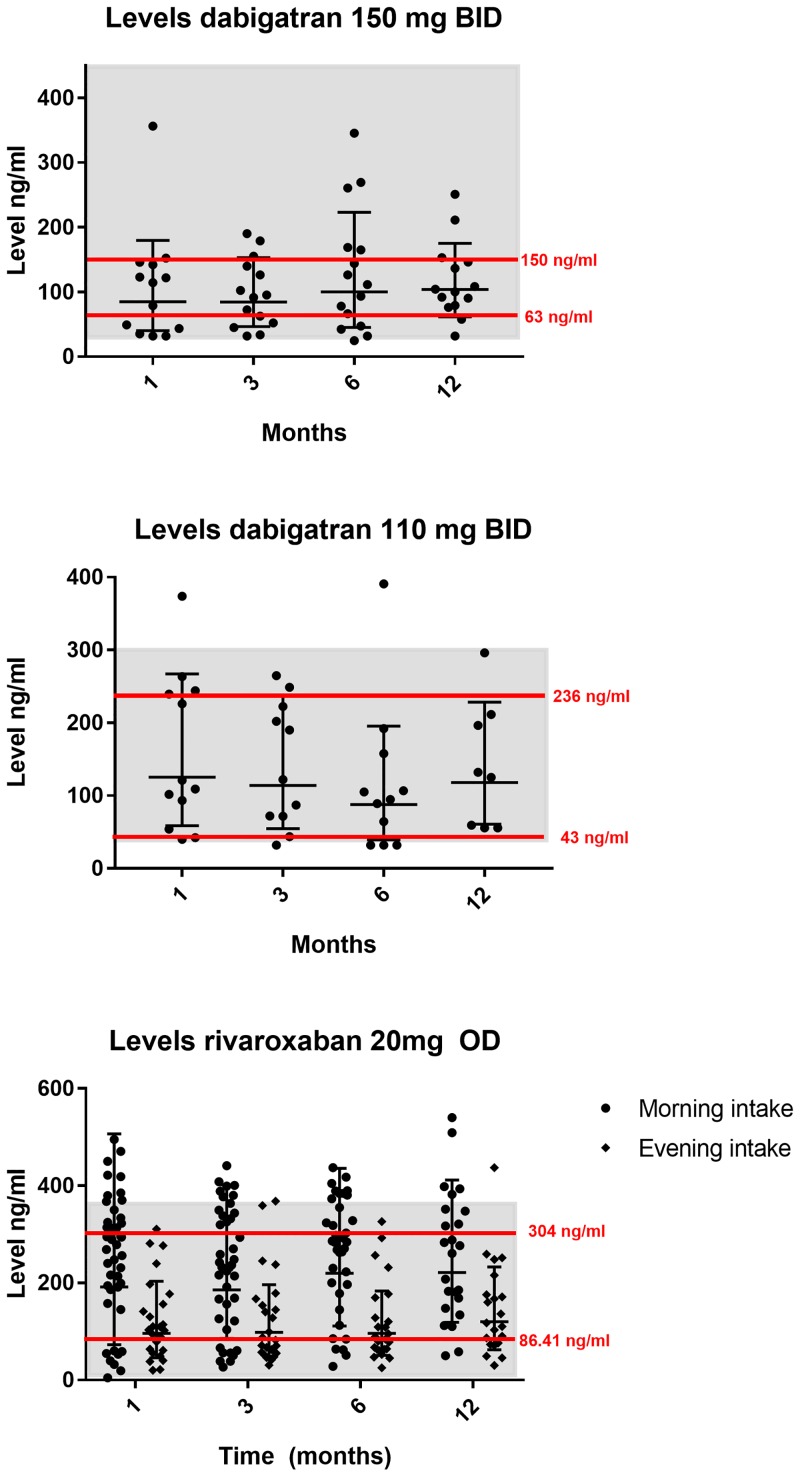
Activity levels DOACs at different time points. The red lines represent the calculated upper and lower 20th percentiles for the different doses. The grey shaded area represents the ‘expected on therapy’ activity levels.

Fig 2 shows trajectories for patients on dabigatran and rivaroxaban respectively who had 2 or more available data points and for whom 1 or more data points were outside the 20^th^ or 80^th^ percentile as shown in Table 2. The grey lines represent patients with more than 1 outlier; the red lines represent patients with 1 outlier.

### Levels of DOAC in relation to outcomes

For dabigatran, 7 seven bleeding events were observed. Bleeding occurred at different points in time and in 7 different patients: 5 bleeding events occurred in patients treated with the 150mg BID dose and 2 in patients treated with the 110mg BID dose. The mean plasma level of dabigatran in patients who experienced a bleeding event was 106.1 ng/ml (SD 71.0) for the 150 mg bd dose. For the 2 patients with a bleeding event treated with a 110 mg bd dose, data of mean plasma levels were only available for 1 of the 2 persons, showing a mean plasma level over time of 231.1 ng/ml (SD 12.0). Since we do not have plasma levels collected at the time of bleeding, it is not possible to correlate actual plasma levels at the time of bleeding with the bleeding events.

For rivaroxaban, in total 10 bleeding events were observed. There were 4 bleeding events in patients with a morning intake: 2 in the 15mg OD dose group (no data available) and 2 in the 20mg OD dose group (mean plasma levels 262.6 ng/ml, SD 112.6). In the patients with a rivaroxaban evening intake, there were 5 bleeding events, all in the 20mg OD dose group. The mean plasma level in this group was 149.4 ng/ml (SD 88.7). In 1 patient data about timing of drug intake was missing.

### Adherence and persistence to DOAC therapy

28 of the 158 patients (17.7%) patients did not persist on initial DOAC therapy and switched to VKA (16), anti-platelet therapy (3), other DOAC or reduced dose same DOAC (5), or stopped anticoagulant therapy altogether (2), and 2 patients switched permanently to LMWH therapy. Of these 28 patients 8 stopped or switched already within the first month of DOAC-therapy. The average time to switch medication was 6 months.

Adherence to DOAC therapy measured at each study visit is visualized in Table 3. Overall, almost 90% of the all patients had a medium adherence score (6-<8). There was a significant association between a history of bleeding and adherence at 1 month (p 0.016), where patients with a bleeding history showed a higher adherence score. However, history of bleeding was not associated with adherence for the entire follow up (p 0.689). Raw data of the adherence measurements are shown (S2 Table).

**Table 3:** Adherence according to Morisky adherence measurement score (MMAS-8).

	High adherence (Morisky = 8) (n,%)	Medium adherence (Morisky = 6-<8) (n,%)	Low adherence (Morisky <6) (n,%)
**1 month (n = 127)**	12 (9%)	110 (87%)	5 (4%)
**3 months (n = 110)**	11 (10%)	95 (86%)	4 (4%)
**6 months (n = 102)**	13 (13%)	86 (84%)	3 (3%)
**12 months (n = 72)**	6 (8%)	66 (92%)	0 (0%)

Note: Use of the MMAS is protected by US Copyright laws. Permission for use is required. A license agreement is available from MMAS Research LLC 14725 NE 20th St. Bellevue WA 98007 or from dmorisky@gmail.com.

There was also no significant association between prospectively recorded bleeding events and adherence score during follow up (p 0.182). Moreover, none of the other patient characteristics such as age, sex, CHA_2_DS_2_-VASc [17], use of NSAID or antiplatelet therapy, malignancy or HASBLED were associated with the adherence score.

### Bleeding and thrombotic events

In total 17 bleeding events were registered during the 1 year follow-up period; this included one major (0.6 major bleeding events /100 patient years) and 16 minor bleeding events (10 minor bleeding events/ 100 patient years) (Table 4) shows the characteristics for the bleeding events.

**Table 4:** Type and severity of bleeding events.

Bleeding type	N
Major bleeds	1
Minor bleeds	16
Digestive tract	2
Urogenital tract	4
Hematoma	6
Nose bleed	1
Conjunctival bleed	2
Traumatic bleed	1

In the univariate analyses only previous bleeding reached a p value of <0.2 (p 0.174) for the association to any bleeding event. None of the tested items such as HASBLED ≥3, malignancy, age≥75 years, gender, drug use (NSAID/anti-platelet) or mild renal failure (≤50 ml/min) reached a p value of <0.2. No thromboembolic events were observed during follow up.

### Prescribed dose of DOAC

Physicians’ prescription behavior for dabigatran and rivaroxaban was analyzed and checked against the recommendations stated in current guidelines.

**Fig 3:**
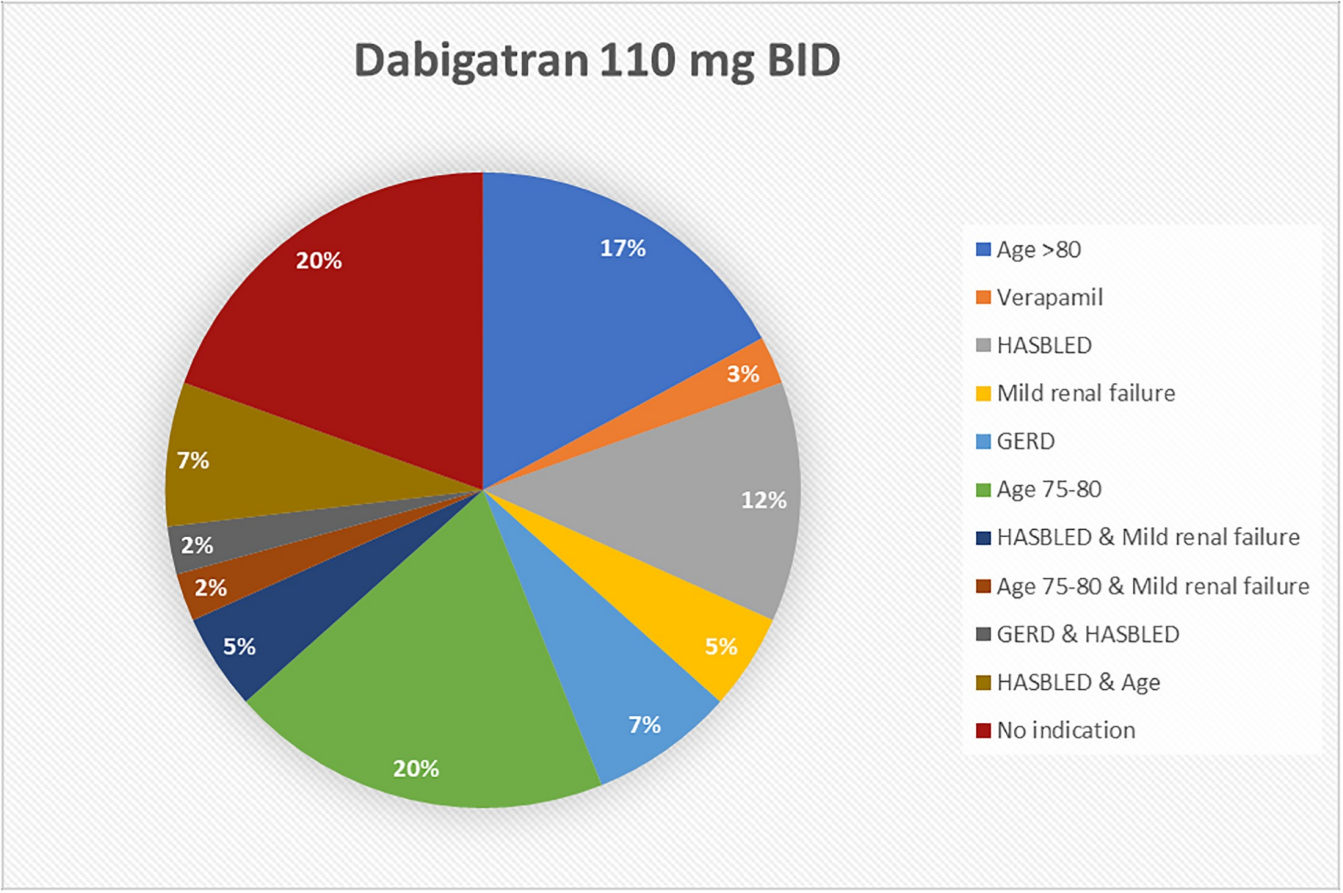
Reasons used by attending physicians to adjust dabigatran dose.

Data on the reasons for dose adjustment for dabigatran are summarized in Fig 3. 41 of 100 (41%) patients were treated with an adjusted dose of dabigatran (110 mg bid). For eight of these patients (19.5%) there were strict recommendations (age>80 or concomitant use of verapamil) for the adjusted dose. More patients had a ‘considerable recommendation’: a HASBLED score ≥3 without correctable risk factors in 11 of 41(26%), mild renal failure in 5 of 41 (12.2%), gastrointestinal disease in 4 of 41 (9.8%) and advanced age in 12 of 41 patients (29.3%). Seven out of 41 (17.1%) patients who were treated with an adjusted dose of dabigatran had more than one reason to lower the dose. Eight patients (19.5%) had no indication for the adjusted dose.

Data on dose adjustment for rivaroxaban showed that twenty-eight of 195 (14.4%) patients were treated with adjusted dose rivaroxaban (15 mg od). In 13 of 28 patients (46.4%) mild renal failure was observed, while renal function was normal in 10 of 28 patients (35.7%). For 5 patients data on renal function were missing.

## Discussion

The focus of this observational cohort study was to assess the stability of DOAC plasma levels over time in relation to patient characteristics, dosing and adherence to medication. The reason for exploring this aspect was our initial concern that in day-to-day care in elderly subjects, due to variations in drug intake, drug interactions or comorbidity, the acclaimed pharmacokinetic stability of DOAC might be compromised.

In addition, the determination of DOAC plasma levels also provides an opportunity to verify the consequence of adjusted dosing schemes. In 41% of patients using dabigatran and 14.4% of patients using rivaroxaban reduced treatment doses were prescribed. Both for dabigatran and rivaroxaban the observed plasma levels were not statistically different for the standard versus the reduced doses of the drugs. Our daily practice findings are in line with previously published data from the RE-LY [18] and ROCKET-AF [19] trials and support the appropriateness of the decision by the cardiologist to adjust the dose in the majority of cases. The most logical explanation would be that impaired renal function hampers adequate drug excretion and, therefore, similar drug plasma levels are observed in patients with reduced doses of the drug. We observed that in the majority of cases prescribing physicians used an adjusted DOAC dose for good reasons. Only in 0.8% (8/100) of dabigatran prescriptions and in 0.5% (10/195) of rivaroxaban treatment no good arguments for a lowered dose were present. This observation is in contrast with recent observational data showing that physicians in daily care consider individual patient characteristics beyond the recommendations for their selection of DOAC dosage much more frequent, in up to 20% of cases[20–23]. Although our study is relatively small, the observed plasma levels consistency among doses suggests that prescriber’s dose selection may often be appropriate.

Over time, there were no significant variations in plasma dabigatran and rivaroxaban levels within patients. However, there were marked variations seen between patients, as was also described in previous pharmacokinetic studies[24, 25]. When exploring the extreme values defined as values in the upper and lower 20^th^ percentiles, it appeared that a significant proportion of patients started within these extreme values and remained there during the one year follow up. Although we showed no direct relationship between negative outcome events and extreme values; this offers a window of opportunity to adjust dose or switch to a different DOAC early after treatment initiation in patients with extreme values to enhance safety and efficacy of NOAC treatment. However, this notion needs validation in a randomized clinical trial comparing fixed dose with plasma level adjusted NOAC treatment.

Adherence to therapy 1 year after start of DOAC treatment was reasonably good with MMAS scores of 6–8 (medium adherence) in 92% and MMAS scores of 8 (good adherence) in 8% of patients. This finding is in contrast to other studies showing a lack of persistence in up to 40% of patients as soon as 3 months of therapy initiation [26, 27], but in line with some earlier reports from DOAC studies in daily care [22, 28]. Three factors may explain the observed adherence rate in our study: first, the fact that the vast majority of patients were previous VKA users, thus trained and supervised in anticoagulant intake; second, study patients knew that their plasma levels were measured which may have stimulated proper intake. Third, personal recruitment for the MMAS-8 questionnaire may have induced a social desirability bias.

Although this study is too small to notice any effect of adherence on outcomes, our data indicate that in this selected and adherent patient population dabigatran and rivaroxaban are well tolerated.

We did not observe any thromboembolic events. Furthermore, the rate of bleeding complications was lower than expected compared to other prospective clinical trials [23, 29]. The low incidence of clinically relevant bleeding and the absence of ischemic events may in part relate to the reasonably good drug intake. From recent studies, it emerges that lack of adherence is indeed associated with an increased risk of ischemic stroke in patients with NVAF[30, 31].

Some limitations of our study need to be mentioned. The observational character of the study may have induced bias and the results of this study concerning adherence may therefore not be generalizable to all patients in day-to-day care. However, this does not negate our pharmacokinetic findings. Patients with the highest risk for bleeding may have been underrepresented in our study sample as almost 90% of patients previously used VKA and were therefore not anticoagulation naïve. This may be an important determinant of the relatively low risk for bleeding in our study population. Furthermore, treatment strategies were not randomly allocated and, therefore, preferential treatment allocation may have influenced study outcomes. The order of introduction of the DOACs on the Dutch market did have an impact on the prescription of DOACs over time, which explains why patients using apixaban or edoxaban were underrepresented in this cohort.

## Conclusion

Under conditions of reasonably good adherence the majority of patients in this observational study remained within therapeutic on-therapy plasma levels and experienced no thromboembolic and relatively few bleeding events. While our data support the assumption that frequent monitoring of DOACs in patients with NVAF is not required, one could argue that initial dose response evaluation may be of benefit in providing an individual patient’s “benchmark” for a particular type and dose of DOAC. Given the range of choices in DOACs (or VKA), optimization of drug and dose selection is feasible and might not be irrelevant.

## Supporting information

S1 Table (PDF)

S2 Table (PDF)
