# Supplementary material for: The daily practice of direct oral anticoagulant use in patients with atrial fibrillation; an observational cohort study
Source: PLoS One. 2019 Jun 6;14(6):e0217302. doi: 10.1371/journal.pone.0217302 (PMC6554016; doi:10.1371/journal.pone.0217302)
Supplement: S1 Table — (PDF) [file pone.0217302.s001.pdf]

Dabigatran 150 mg bid				Dabigatran 110mg bid			
**1 Month**	**3 Month**	**6 Months**	**12 Months**	**1 Month**	**3 Month**	**6 Months**	**12 Months**
		42,4			72		
	95,4	269,6	251,1	226	202	106,8	
		168,9	104,4	239,6	222,6		
32	33,9	32		42,4	43,8	32	55,7
32	45,2	144,3	32	373,9	264,7	390,8	55,7
142,4	91,5	25	136,6			32	59,6
			57,9	109,2			
	32				248,8	64,5	132,3
43,8	62,8	93,4			190,3	192	211,5
		111,6	100,3	54	71,9	94,9	
		66,4	211,2	244,2			
152,5	155,3			101,7		157,7	296,3
122,1				263,5			
123	179,2			93,4	87,2	105,1	
49,4	72,7	47,3	91,9	40,2	32	32	
79,1	52,2	78,2	90,5			89	125,1
			76,2	121,3	122,1		
356,3	190,3	345,6					196,5
146	102,5						
		126,4	79,3				
			153,3				
114,5	126,4	260,9	108,5				
35,3	139,9	165,1					

Rivaroxaban 20 mg combined			
**1 Month**	**3 Month**	**6 Months**	**12 Months**
159,6	208,1	285,8	
		230,1	289,8
9	18,6	301,8	101,9
117,8			
	375,4		
384,8	319,4	306	
	23		
	122,3	86,5	81
178,7			
		52,3	44,5
		292,6	540,4
	38,6	284,3	50
256,3	293,6		
194,1	214,2	262,5	168,6
278,8	216,2	283,7	
157,7	166,5		
385,4	380	384,1	
	399	373,2	58,3
	319,7	144,9	110,1
	248	200,3	276,6
312,8	363,9	389,5	260,8
191,3		196,7	
470,5			
350,4			
421,6	408	384	
370,2	270,1	302,7	398,2
379,8	242,3	404,7	
247,5	26,7	230,3	283,3
	332	297,9	
300,2	325,8	328,2	
216	55,8	51,4	
231,4	191,9		
199,2			
			134,3
324,8	338,3		185,2
322,9	231,1	263,9	112,2
450	441,4	417,9	509,3
58,7	50,5	112,5	181,1
268,7	121,8		
289,3		323,7	321,1
32	388,7		
294,8	400,8	380,6	288
240,3	103,6	318,3	
313,4	232,1		
60,1	60,9	268,2	317,1
495,1			
294	66	286,1	
186,4			

	126,3	62,4	147,6
39,8	38,9	28,3	
367,8	229	85,1	351,7
314,4		355	
54,6	168,9	178	
19,2	224,2	222,7	
53,2	258,6	389	393,8
295,6			
333,8	377	437,1	382,3
418,9	343,7	63,8	207,5
4,7	56,3	84,2	
	156,4	270,9	347,6
145,2	349,7		
213,6	236,6		
			182,9
89,2	71,2		73,1
276,8			
239,7	167,2	256,5	
63,7	46,3		117,7
197	178,8	128	
	47,7	63	86,8
	99,2	63,5	
61,3	55,4	47,1	
78,5		120,1	171,3
103	368,1	108,7	160,3
281,3	359,1		
102	87,6	80,3	103,3
94,2	153,7	109,3	167,1
141,1	57,2	89,4	
55,1	128,3	84	136,3
156			
		292,5	251,6
			259,1
310,5		326,3	248,3
	70,6	67,1	71
49,2	62,5	64,3	76,4
20,3	30,9	60,1	45,8
177,1	237,7	231,6	215,9
	82	78,4	30,4
130,2	144,5		
110,3	245,3	95,3	437,3
109,7	140,2	52,8	
		169,5	110,3
			90,3
40	41,6	45,1	
21,8	52,1	25,1	
38	67,3		49,1
114,4		177,2	175,9
