# Supplementary material for: The daily practice of direct oral anticoagulant use in patients with atrial fibrillation; an observational cohort study
Source: PLoS One. 2019 Jun 6;14(6):e0217302. doi: 10.1371/journal.pone.0217302 (PMC6554016; doi:10.1371/journal.pone.0217302)
Supplement: S2 Table — (PDF) [file pone.0217302.s002.pdf]

Month 1	Month 3	Month 6	Month 12
7	7		
7	7	8	
5			
7	7	7	7
7	7	7	7
7	7	7	7
7	7	7	
7			
8	8	8	
7	7	7	
8	6	5	8
5	5	7	6
7	7	8	7
7	7	8	7
		7	
	7	7	7
	7	7	
7	7	7	7
6	7	7	7
8	7	7	7
7	6	7	
7	7	7	
7	7	7	
7			
7	7	7	7
7	7	7	7
7	7	7	
7		7	8
8	7		
7	7	7	7
7	8	8	7
7	7	8	7
7		7	7
7	5	7	
7	7	7	
7	7	7	
7	7	7	7
7	7	7	
7	7	7	
7			
7	7	7	7
7	7		
7	7	7	7
		7	7
7	8	7	8
7	6	6	7
7	7	6	7
7		7	

7	7		
7	7	8	7
7	7	8	7
	8	8	7
7	7		
7	7	7	
7			
7	7	7	7
	7		
7	7	8	7
7			
7	7	7	
	7	7	7
7	5	7	
7	7	7	7
7	7	6	7
8	8	7	7
7	7	6	7
7	7	7	
7	8	8	7
7	7	6	7
6	6	7	7
7	7	6	
7	7		7
7		7	7
7	7		
7	7	7	7
8		7	6
7	7	7	
7	7	7	7
8	8	7	8
7	7	5	6
7	7	7	7
8	8	7	7
6			
7	7	7	8
7	7		
7	7	7	7
7	7	7	7
7	7	7	7
	6	7	7
7	7	7	7
5	5	8	7
7	7	7	
	7		
7	7	7	7
7	7	7	
7			
8	8	7	7
	6	5	

7	7		
	7	7	
7	8	8	
7	7	7	8
7	7	7	7
7	7		
8			
7	7	7	7
7	7	7	7
7			
7	7	7	7
6	7		
	6		
7	7	7	7
7	7	7	7
7	7	7	
7	7		
7	7	6	7
7	7	7	7
7	7	7	7
5	7	7	
7	7	7	6
8	7	7	7
5			
7	7	7	
7	7	7	
6	7	7	7
6	7	7	7
6	6	7	7
8	8	7	7
